# Effect of tobacco and nicotine in causing staining of dental hard tissues and dental materials: A systematic review and meta‐analysis

**DOI:** 10.1002/cre2.683

**Published:** 2022-11-13

**Authors:** Rijula R. Karanjkar, Philip M. Preshaw, Janice S. Ellis, Richard Holliday

**Affiliations:** ^1^ Newcastle upon Tyne Hospitals NHS Foundation Trust Newcastle upon Tyne UK; ^2^ Restorative Dentistry, School of Dental Sciences, Faculty of Medical Sciences Newcastle University Newcastle upon Tyne UK; ^3^ Restorative Dentistry, School of Dentistry University of Dundee Dundee UK

**Keywords:** discoloration, electronic nicotine delivery system, heated tobacco product, tobacco

## Abstract

**Introduction:**

Dental staining is a common concern for tobacco users. However, little is known about which components of tobacco are responsible for the staining and whether nicotine may be implicated. This is of increasing relevance with the popularity of novel products such as heated‐tobacco products and electronic cigarettes (E‐cigarettes).

**Objectives:**

This systematic review aimed to establish the evidence base for the effect if any, of the various tobacco and nicotine products in causing staining of dental hard tissues and materials.

**Material and Methods:**

This systematic review was performed in accordance with the preferred reporting items for systematic reviews and meta‐analyses guidelines. There were four structured population intervention comparison outcomesquestions. A search was conducted up to December 2021 in three databases: MEDLINE, EMBASE, and Web of Science, and manual searching of relevant sources was also completed. Two researchers individually reviewed the titles then abstracts and finally full articles. A reporting quality appraisal was conducted appropriately to the study methodology.

**Results:**

Of the 815 records titles identified, 56 full‐text articles were assessed for eligibility, of which 27 were included for analysis. The included studies were mainly laboratory studies of varying reporting quality. There was evidence from 18 studies that tobacco exposure caused staining of dental hard tissues (pooled results from three studies‐ enamel/dentine; mean difference [MD]: 16.22; 95% confidence interval[12.11, 20.32; *I* ^2^: 96%)and materials (pooled result from four studies—resin composite; MD: 11.90; 95% CI: 11.47, 12.34; *I* ^2^: 100%). There was limited evidence that E‐cigarettes 99%) and heated tobacco products (HTPs; pooled results from three studies–−1.07, 6.54; *I* ^2^: 99%) cause staining, but this was lower than with traditional tobacco/found 11 compounds, of which 8 were terpenoids, from tobacco products implicated in causing staining. Finally, there was some evidence that resin composites stained more than other materials.

**Conclusions:**

Tobacco smoking causes dental staining. There was limited evidence that E‐cigarettes and HTPs did cause dental staining that was less intense than that caused by traditional tobacco products.

## 1 INTRODUCTION

Dental discoloration is the alteration of the natural tooth color. It can be classified based on the location of the discoloration or staining, into internal discoloration or external staining (Watts & Addy, 2001). Tobacco smoking has long been considered a cause of dental staining. Reducing the yellowing of teeth, observed in smokers, has often been used as a motivation to quit.

Tooth shade and color changes due to discoloring agents can be difficult to quantify and perceive. The CIELAB color space was introduced in 1976 to better interpret color perception. Change in color ∆E can be calculated (Joiner & Luo, 2017). The minimum change required for a perceptible change, or just a noticeable difference, has been suggested to be between 1.2 and 2.7, and the level of unacceptable change has been suggested to be above 2.7 (Paravina et al., 2019).

Although dental staining in smokers is a common clinical observation, the research evidence in this field has never been formally consolidated. Cigarettes are the most commonly used form of tobacco (World Health Organization, 2020). Novel products such as electronic cigarettes (E‐cigarettes) and heated tobacco products (HTPs) have increasingly become popular. Therefore, in the era of novel tobacco and nicotine products, these questions are much more relevant, as it is unknown which components of tobacco cause staining and what is the true staining potential of the novel products.

E‐cigarettes deliver nicotine within an inhalable aerosol by heating a solution (e‐liquid) of which there are over 7000 identified flavors with varying colors (McEwen & McRobbi, 2016; Zhu et al., 2014). HTPs heat inserted tobacco sticks to a high temperature, just below combustion, releasing aerosols to deliver nicotine (Glantz, 2018).

Smoke directly exuded from a lit cigarette is often described as ‘‘whole smoke,’’ it consists of liquid droplets in aerosol, commonly called the particulate phase, suspended in a mixture of gases and semivolatile compounds. When this is free of nicotine, it is often referred to as nicotine‐free particulate fraction or ‘‘tar’’ (Thielen et al., 2008). This tar collects on cigarette filters turning them yellow–brown suggesting that it is these tar components that stain the dentition (Zanetti et al., 2019). E‐cigarettes produce an aerosol containing nicotine amongst other compounds but do not produce a particulate fraction similar to cigarette smoke. HTPs have also been shown to create less particulate matter than cigarette smoke (Simonavicius et al., 2019). Therefore, a line of argument is that these products, as a result, may cause less staining when compared to conventional smoking.

Manufacturers have often promoted these novel products with claims including, ‘‘no smelly clothes’’ or ‘‘no yellow teeth’’ (Vapex E‐Cigarette, 2020). These are cosmetic rather than health‐based claims and are subject to less stringent regulations. Often smokers are negatively affected by the effect of dental discoloration on their appearance (Alkhatib et al., 2005; Andersson & Johannsen, 2016). The range of cosmetic whitening products available and the popularity of these could suggest that it is a substantial concern for consumers (Eachempati et al., 2018). The lack of evidence supporting the advertising claims leaves room for misrepresentation.

The aim of this systematic review was to establish the evidence base for the effect of the various tobacco and nicotine products in causing staining of dental hard tissues and materials. Specifically:

(1) What effect do tobacco and nicotine products have on the staining of dental hard tissues and materials?
(2) Is there a variation in the levels of staining caused by different tobacco and nicotine products?
(3) Are any particular ingredients identified in tobacco that may cause dental staining?
(4) Is there a variation, in the extent of staining of dental materials, caused by the different tobacco and nicotine products?

## 2 MATERIALS AND METHODS

The systematic review was conducted as per the preferred reporting items for systematic reviews and meta‐analyses checklist (Supporting Information: Table 1), and the protocol was prospectively registered on the PROSPERO database for systematic reviews ID number CRD42018086331 (Karanjkar et al., 2018). Structured population intervention comparison outcomes questions were used to formulate the clinical questions. The population included all laboratory studies with teeth or dental materials and clinical studies with users of tobacco/nicotine in all the various forms. The intervention of interest was tobacco or nicotine exposure in any of their forms. Studies were required to have appropriate negative controls with no tobacco or nicotine exposure. Baseline results for self‐control were accepted. The outcomes of interest were staining of dental hard tissues or dental materials.

### 2.1 Search strategy

A comprehensive search was conducted of the databases Ovid MEDLINE, EMBASE, and Web of Science up to and including December 2021. The same keywords and combinations were used for all databases (Supporting Information: Table 2). Hand searching of online journals from 1980 to December 2021 was completed for the *Journal of Clinical Periodontology, Journal of Periodontology, Journal of Periodontal Research, Periodontology 2000, Dental Materials*, and *Journal of Dental Research. International Association of Dental Research* abstracts from the last 5 years were searched and trial registers *European Clinical Trials Register, the World Health Organisation International Clinical Trials Registry Platform, US National Library of Medicine Clinical Trials*, and *UK Clinical Trials Gateway* were screened for any ongoing studies up to December 2021. Bibliographies of review articles, relevant studies, and two seminal textbooks (Lindhe et al., 2015; Newman et al., 2014) were also reviewed for any relevant studies.

### 2.2 Study selection

All in vivo and in vitro studies with suitable nonexposure control or comparison between types of exposures or comparison between different types of materials were included. Review articles, case reports, and case series were excluded. Any studies comparing dental materials that were older than 25 years were excluded, where the materials were not relevant to current clinical practice. Nonenglish abstracts were reviewed, and an attempt was made to translate them if they were felt to be relevant at the full‐text review stage.

Titles and abstracts were screened by two independent reviewers (Rijula R. Karanjkar and Richard Holliday). Full texts of eligible studies were reviewed against the inclusion and exclusion criterion and any discrepancies were resolved by discussion or by consulting a third reviewer (Philip M. Preshaw). Relevant data were then extracted from the full texts by Rijula R. Karanjkar and Richard Holliday independently using a purposely designed data extraction form and discrepancies were resolved as described (Supporting Information: Table 3).

### 2.3 Reporting quality appraisal

An assessment of reporting quality was completed for the included studies. A 15‐item modified consolidated standards of reporting trials (CONSORT) checklist (Faggion, 2012) was used to give a score out of 15 on all laboratory studies. The strengthening of the reporting of observational studies in the epidemiology checklist (von Elm et al., 2014) was used to give a score out of 22 for any nonrandomized clinical studies. Finally, the CONSORT checklist (Schulz et al., 2010) was used to give a score out of 25 for any randomized control trials.

### 2.4 Data synthesis

Information from data collection sheets and reflection on reporting quality assessments were used to determine the suitability of studies for possible data synthesis and quality of studies. Due to the variation in the study type and design included, a meta‐analysis of all the studies was not possible. Care was taken to ensure that data were pooled only from those studies with comparable study designs. Reported mean change in color (∆E) and standard deviation based on the CIELab formula were the basis for any possible quantitative data analysis. A descriptive narrative synthesis of the findings from the included studies, structured around the four review questions was completed. Where appropriate, data from studies were pooled using a random‐effects meta‐analysis model using RevMan (Version 4) software, and sub‐group analysis was completed to assess differences between different tobacco and nicotine products.

## 3 RESULTS

### 3.1 Study search

**Figure 1:**
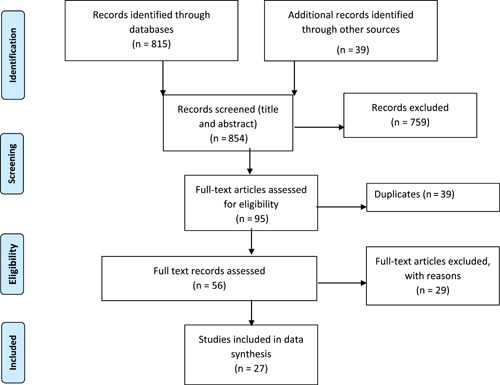
Preferred reporting items for systematic reviews and meta‐analyses flowchart.

The flowchart of the manuscripts screened is shown in Figure 1. A total of 27 studies were included in the full data analysis, after the exclusion of 29 studies. Supporting Information: Table 4 provides reasons for the exclusion of reviewed full‐text studies. A summary of the study characteristics of the included studies is shown in Supporting Information: Table 5. Out of the 27 full texts, 24studies were in vitro laboratory studies, 2 were epidemiological studies, and 1 was a randomized control trial. Table 1 shows key elements of included studies in groups.

**Table 1:** Summary of included studies based on the aims of the review

Aim	Effect of tobacco or nicotine products on the staining of dental hard tissues and materials			
	Outcome assessed	Study	Study type	Included in meta‐analysis
	Effect of cigarette smoke/combusted tobacco particulate on enamel/dentine	Alkhatib et al. (2005)	Observational study	No
		Dalrymple et al. (2018)	In vitro study	Yes
		Haiduc et al. (2020)	In vitro study	Yes
		Ness et al. (1977)	Observational study	No
		Amorim et al. (2021)	In vitro study	No
		Dalrymple et al. (2021)	In vitro study	Yes
		Kobayashi et al. (2021)	In vitro study	No
	Effect of nicotine products on enamel/dentine:			
	‐E‐cigarettes	Dalrymple et al. (2018, 2021)	In vitro study	Yes
	‐Nicotine chewing gum	Moore et al. (2008)	In vitro study	No
	‐Nicotine pouches	Dalrymple et al. (2021)	In vitro study	Yes
	Effect of heated tobacco product on enamel/dentine	Dalrymple et al. (2018)	In vitro study	Yes
		Haiduc et al. (2020)	In vitro study	Yes
		Dalrymple et al. (2021)	In vitro study	Yes
	Effect of smokeless tobacco product on enamel/dentine	Dalrymple et al. (2021)	In vitro study	Yes
	Effect of cigarette smoke on resin composite	Taraboanta et al. (2019)	In vitro study	Yes
		Belli et al. (1997)	In vitro study	No
		Mathias, Costa et al. (2010)	In vitro study	Yes
		Mathias et al. (2011)	In vitro study	Yes
		Vohra et al. (2020)	In vitro study	Yes
	Effect of E‐cigarettes on resin composite	Vohra et al. (2020, 2020)	In vitro study	Yes
	Effect of cigarette smoke on ceramic	Belli et al. (1997)	In vitro study	No
		Vohra et al. (2020)	In vitro study	Yes
		Ayaz et al. (2014)	In vitro study	Yes
	Effect of E‐cigarette on ceramic	Vohra et al. (2020)	In vitro study	Yes
	Effect of cigarette smoke on acrylic	Ayaz et al. (2014)	In vitro study	Yes
		Patil et al. (2013)	In vitro study	Yes
		Wang et al. (2022)	In vitro study	Yes
	Effect of heated tobacco product on acrylic	Wang et al. (2022)	In vitro study	Yes

Aim	Comparison between the effect of different tobacco or nicotine products on dental staining			
	Outcome assessed	Studies (reference)	Study type	Included in meta‐analysis
	Effect of cigarette smoke and E‐cigarettes on dental staining	Dalrymple et al. (2018)	In vitro study	Yes
		Dalrymple et al. (2021)	In vitro study	Yes
		Vohra et al. (2020)	In vitro study	Yes
		Zhao et al. (2019)	In vitro study	Yes
	Effect of cigarette smoke and heated tobacco product on dental staining	Zanetti et al. (2019)	In vitro study	Yes
		Dalrymple et al. (2018)	In vitro study	Yes
		Haiduc et al. (2020)	In vitro study	Yes
		Dalrymple et al. (2021)	In vitro study	Yes
		Zhao et al. (2017)	In vitro study	Yes
	Effect of heated tobacco product and E‐cigarette on dental staining	Dalrymple et al. (2018)	In vitro study	Yes
		Dalrymple et al. (2021)	In vitro study	Yes
	Effect of different types of nicotine replacement therapies on dental staining	Moore et al. (2008)	In vitro study	No
		Whelton et al. (2012)	Randomized controlled trial	No
	Effect of different E‐cigarette flavors on dental staining	Dalrymple et al. (2021)	In vitro study	No
		Pintado‐Palomino et al. (2019)	In vitro study	No
	Effect of different nicotine concentrations on dental staining	Pintado‐Palomino et al. (2019)	In vitro study	No
		Lertsukprasert et al. (2020)	In vitro study	No
	Effect of smokeless tobacco (snus), pouches, and cigarette smoke on dental staining	Dalrymple et al. (2021)	In vitro study	No

Aim	Specific components of tobacco that cause dental staining			
	Outcome assessed	Studies (reference)	Study type	Included in meta‐analysis
	Identifies components that cause dental staining	Haiduc et al. (2020)	In vitro study	Not applicable

Aim	Comparison in levels of staining of dental substrates due to tobacco and nicotine products			
	Outcome assessed	Studies (reference)	Study type	Included in meta‐analysis
	Comparison in the level of staining of enamel/dentine and resin composite	Zanetti et al. (2019)	In vitro study	No
		Zhao et al. (2019)	In vitro study	No
	Comparison in the level of staining of types of resin composite	Taraboanta et al. (2019)	In vitro study	No
		Belli et al. (1997)	In vitro study	No
		Mathias, Costa et al. (2010)	In vitro study	No
		Malhotra et al. (2011)	In vitro study	No
		Mathias, Silva et al. (2010)	In vitro study	No
		Theobaldo et al. (2020)	In vitro study	No
		Wasilewski et al. (2010)	In vitro study	No
		Alandia‐Roman et al. (2013)	In vitro study	No
	Comparison in the level of staining of resin composite and ceramic	Belli et al. (1997)	In vitro study	No
		Vohra et al. (2020)	In vitro study	No
	Comparison in the level of staining of ceramic and acrylic	Ayaz et al. (2014)	In vitro study	No

Abbreviation: E‐cigarette, electronic cigarette.

### 3.2 Reporting quality assessment and heterogeneity of studies

The results of the studies reporting quality assessments are shown in Supporting Information: Table 6. Most of the in vitro studies did not explain how sample size was calculated or include details of randomization or blinding. Often there was an incomplete discussion on limitations and sources of bias. Many studies were brief on the details of their methods and some had missing information on funding sources.

There were differences in study methods between the in vitro laboratory studies such as differences in product types tested, controls used, instrumentation used to measure color change, presence or absence of brushing between exposures, and the timing that color change readings were taken. However, many of these studies assessed the change in color of substrates as ∆E, as opposed to the exact color, according to the CIELab system. As this measure assesses the relative change, it was acceptable to use it as a parameter for combining information from studies. The other parameter that made collating of data acceptable was the presence of an appropriate control that was comparable between studies. As 24 out of 27 studies included were in vitro studies, we proceeded with a pragmatic approach utilizing the reporting quality assessment to explore the quality of studies. A formal risk of bias assessment was not completed.

### 3.3 Effect of tobacco or nicotine products on the staining of dental hard tissues and materials

Eighteen studies explored the effect of tobacco or nicotine products on the staining of dental enamel, dentine, resin composite, dental ceramic porcelain, or acrylic (Supporting Information: Table 5).

#### 3.3.1 Effect on enamel or dentine

**Figure 2:**
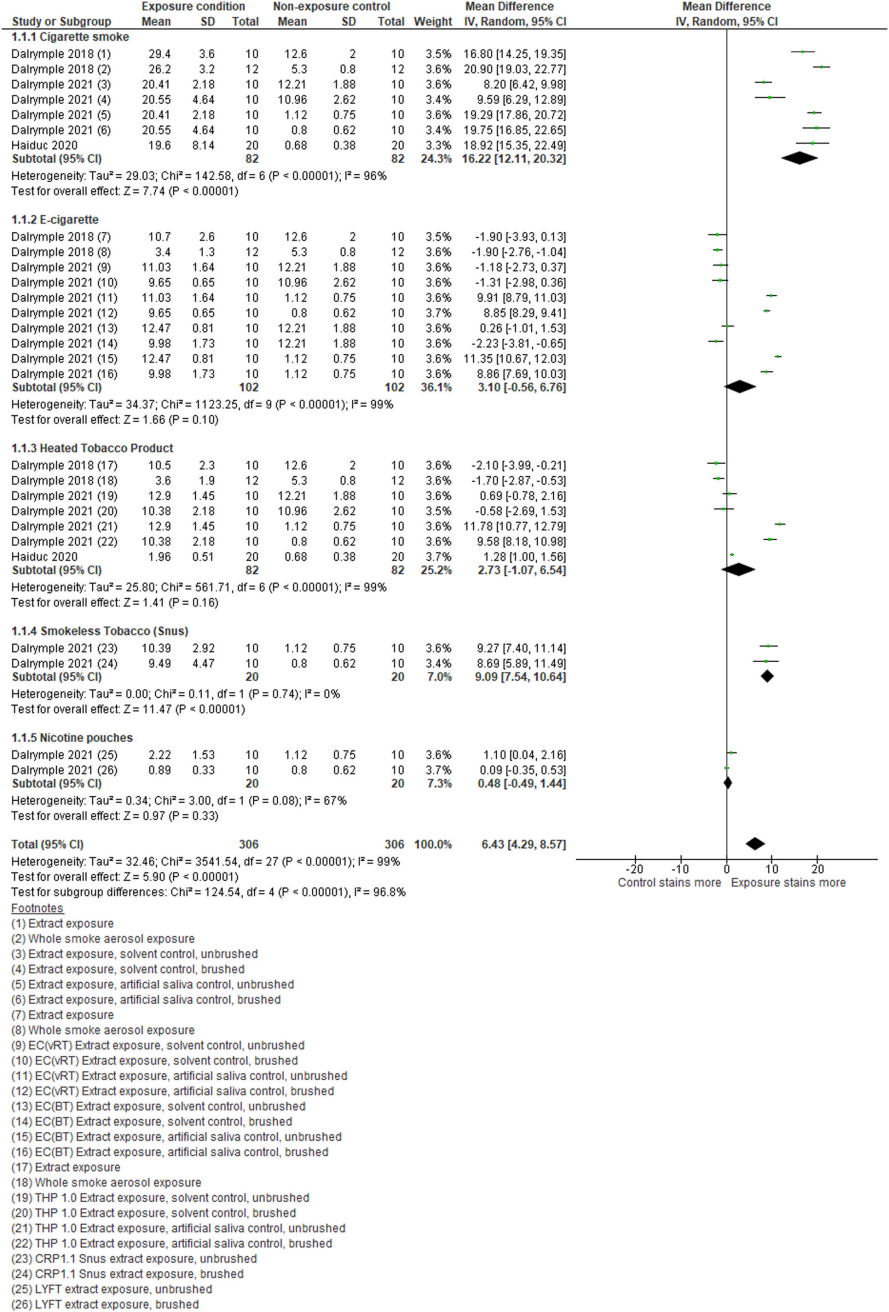
Forest plot for subgroup analysis of discoloration of enamel from exposure to cigarette smoke/extract, E‐cigarette aerosol, and heated tobacco product aerosol compared with a nonexposure control. CI, confidence interval; CRP, C‐reactive protein; E‐cigarette, electronic cigarette; SD, standard deviation; THP, tetrahydropyran.

Seven studies assessed the effect of cigarette smoke/combusted tobacco particulate (CS extract) exposure on enamel or dentine (Alkhatib et al., 2005; Amorim et al., 2021; Dalrymple et al., 2018, 2021; Haiduc et al., 2020; Kobayashi et al., 2021; Ness et al., 1977). We pooled three studies with similar methodologies (Dalrymple et al., 2018, 2021; Haiduc et al., 2020) and found evidence that cigarette exposure caused a large change in color (increased staining), measured by ∆E, of enamel or dentine compared with a nonexposure control (mean difference [MD]: 16.22; 95% confidence interval [CI]: 12.11, 20.32; *I* ^2^: 96%; Figure 2). Within the studies not included in the meta‐analysis, Amorim et al. (2021) showed that cigarette smoke caused a much smaller change in color (CIELab E00) if compared to the mean difference, this was still above the limit of perceptibility and acceptability. However, Kobayashi et al. (2021) showed that when estimated, cigarette smoke caused twice the color change (CIELab ∆E) of a nonexposure control. The remaining two studies (Alkhatib et al., 2005; Ness et al., 1977) comparing smokers with nonsmokers showed a significant association between smokers experiencing greater moderate to severe discoloration when compared with nonsmokers (Supporting Information: Table 5).

With reference to nicotine products, two studies assessed the effect of E‐cigarettes on enamel or dentine. Dalrymple et al. (2018) included two datasets, one assessing the exposure of E‐cigarette particulate matter extract exposure and the other of E‐cigarette aerosol exposure to enamel substrates comparing them to nonexposure controls. Dalrymple et al. (2021) included data on two different E‐cigarette extract exposures, comparing them with artificial saliva or solvent control. These were further subdivided into brushed or unbrushed subsets, resulting in eight datasets. Data from the resultant 10 datasets in these studies (Dalrymple et al., 2018, 2021) were pooled, and evidence was found that E‐cigarette exposure changed the color of enamel more than that of the non‐exposure control (MD: 3.10; 95% CI: −0.56, 6.76; *I* ^2^: 99%; Figure 2).

One study (Moore et al., 2008) assessed the effect of two different nicotine chewing gums and a whitening confectionery gum with a saliva control. This study assessed ∆E stain reduction after the use of the gum and found that the two nicotine gums removed more stain compared to the whitening gum and control.

One study (Dalrymple et al., 2021), with two datasets, assessed the effect of nicotine pouches. Pooled results show it caused minimally more stains than the artificial saliva control (MD: 0.48; 95% CI: −0.49, 1.44; *I* ^2^: 67%; Figure 2).

Three studies assessed the effect of HTPs on enamel and dentine (Dalrymple et al., 2018, 2021; Haiduc et al., 2020). Data from these studies were pooled and evidence was found that exposure to HTPs caused more staining when compared to a nonexposure control (MD: 2.73; 95% CI: −1.07, 6.54; *I* ^2^:99%; Figure 2).

One study (Dalrymple et al., 2021) with two datasets, assessed the effect of smokeless tobacco (snus). Pooled results show it caused more stains than the artificial saliva control (MD: 9.09; 95% CI: 7.54, 10.64; *I* ^2^: 0%; Figure 2).

#### 3.3.2 Effect on resin composite

**Figure 3:**
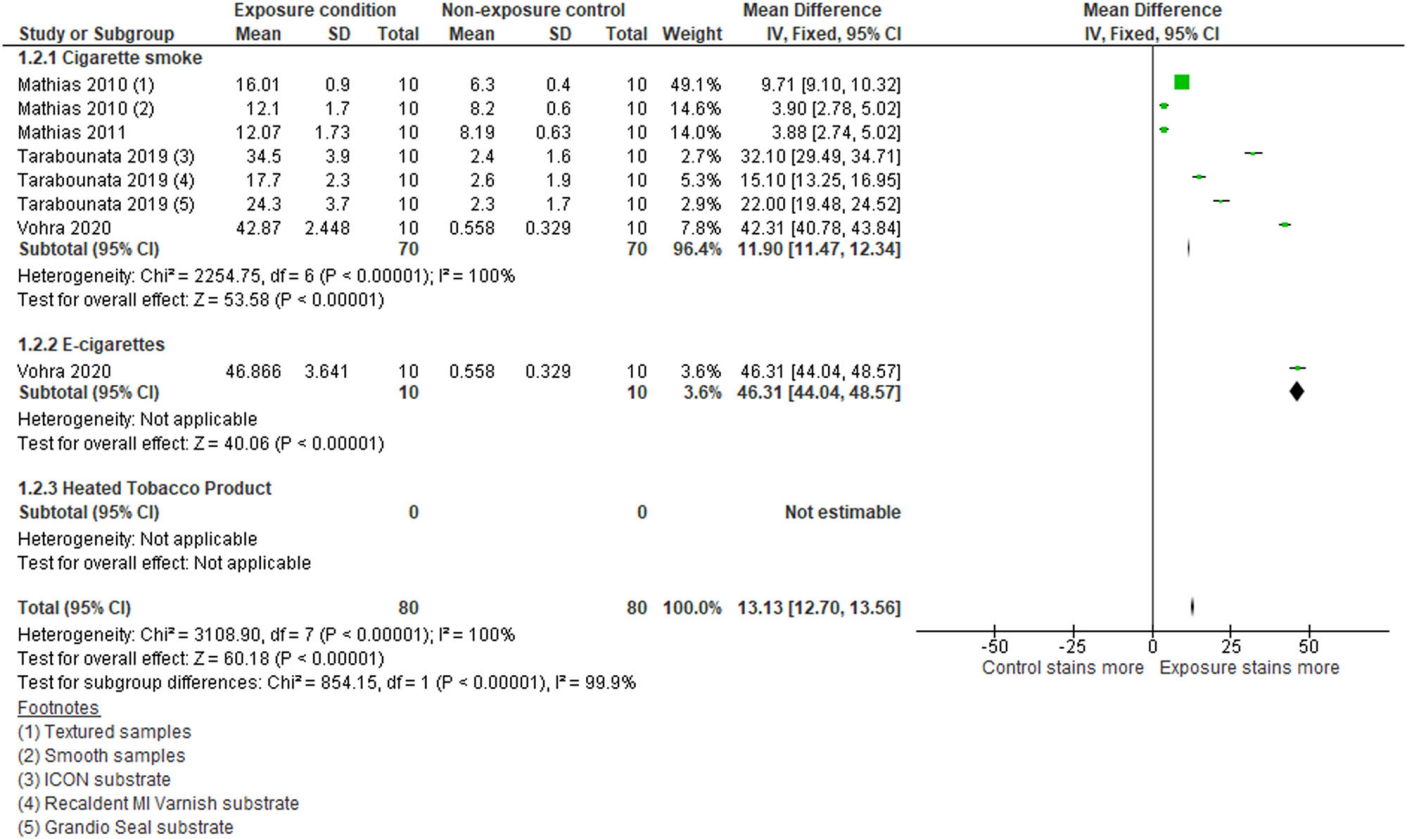
Forest plot for subgroup analysis of discoloration of resin composite from exposure to cigarette smoke/extract and E‐cigarette aerosol compared with a nonexposure control. CI, confidence interval; E‐cigarette, electronic cigarette; SD, standard deviation.

Five studies assessed the effect of cigarette smoke on resin composite (Figure 3) (Belli et al., 1997; Mathias, Costa, et al., 2010; Mathias et al., 2011; Taraboanta et al., 2019; Vohra et al., 2020). Data from four studies were pooled (Mathias, Costa, et al., 2010; Mathias et al., 2011; Taraboanta et al., 2019; Vohra et al., 2020) two of which (Mathias, Costa, et al., 2010; Taraboanta et al., 2019) had subgroups of resin composite materials, into a dataset and found evidence that cigarette smoke causes increased staining of resin composite materials when compared to a non‐smoker exposure control of (MD: 11.90; 95% CI: 11.47, 12.34; *I* ^2^: 100%). The study not included in the meta‐analysis, Belli et al. (1997), also showed evidence of an increase in staining in two resin composites tested upon exposure to cigarette smoke versus a distilled water control (Supporting Information: Table 5).

With regard to E‐cigarettes, Vohra et al. (2020) explored the effect of E‐cigarette exposure, compared with a no‐smoke artificial saliva control, on resin composite (Figure 3) and showed evidence of an increase in staining when compared with the control (MD: 46.31; 95% CI: 44.04, 48.57). These results were like the effect seen when samples were exposed to cigarette smoke (MD: 42; 95% CI: 40.78, 43.84)

In relation to HTPs, none of the studies assessed the effect of an HTP aerosol exposure compared with a no‐smoke exposure control on resin composite.

#### 3.3.3 Effect on dental ceramic

Three studies assessed the effect of cigarette smoke on ceramics (Ayaz et al., 2014; Belli et al., 1997; Vohra et al., 2020). Data from two studies were pooled (Ayaz et al., 2014; Vohra et al., 2020) exploring the effect of cigarette smoke compared with a nonexposure control on dental ceramics. There was evidence that cigarette smoke caused slightly more staining on ceramic in comparison to the control (MD: 1.53; 95% CI: 1.44, 1.62; *I* ^2^: 83%, Supporting Information: Figure 1). The study not included in the meta‐analysis, Belli et al. (1997) found evidence that cigarette smoke upon exposure caused discoloration of ceramic (Supporting Information: Table 5).

With regard to E‐cigarettes, Vohra et al. (2020) found evidence that E‐cigarette aerosol exposure caused an increase in the staining of dental ceramic compared to nonsmoke control (MD: 2.10; 95% CI: 1.71, 2.50; Supporting Information: Figure 1).

None of the studies assessed the effect of heated tobacco product aerosol exposure compared with a no‐smoke exposure control on dental ceramics.

#### 3.3.4 Effect on acrylic

Data from three studies were pooled (Ayaz et al., 2014; Patil et al., 2013; Wang et al., 2022) comparing the effect of cigarette smoke, to a no‐smoke exposure, on six different commonly used acrylics and found evidence of an increase in staining of acrylics (MD: 5.86; 95% CI: 5.71, 6.00; *I* ^2^: 100%; Supporting Information: Figure 2).

When considering E‐cigarettes and HTPs, Wang et al. (2022) found evidence that HTP aerosol exposure caused a minimal increase in staining of dental acrylic compared to nonsmoke control (MD: 0.45; 95% CI: 0.36, 0.54; Supporting Information: Figure 2). None of the studies assessed the effect of E‐cigarettes compared with a no‐smoke exposure to acrylic.

### 3.4 Comparison between the effect of different tobacco or nicotine products on dental staining

#### 3.4.1 E‐cigarette and cigarette smoke

**Figure 4:**
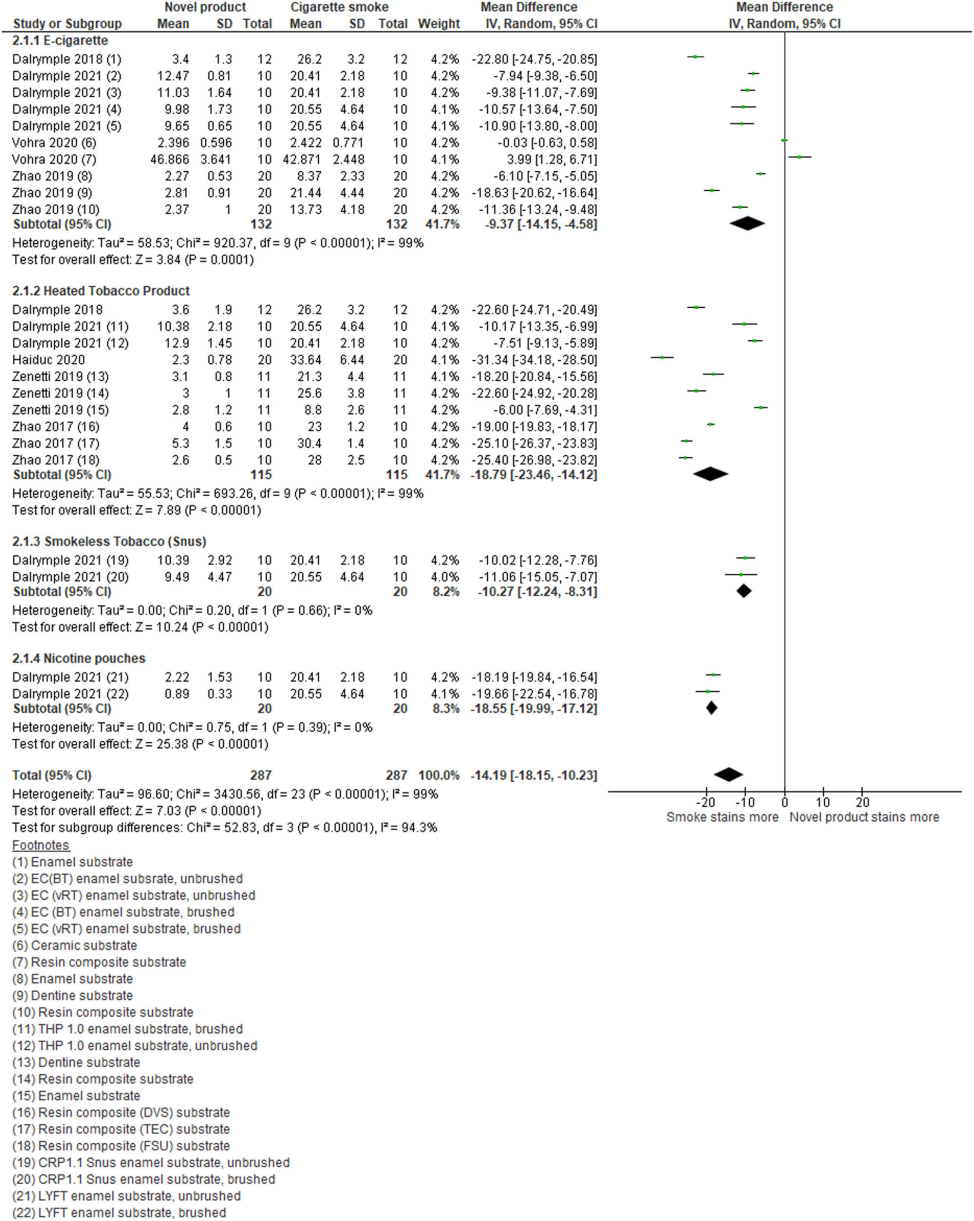
Forest plot of subgroup analysis for comparison of the novel product (E‐cigarette and heated tobacco product) aerosol and cigarette smoke/extract exposure on enamel, dentine, and resin composite. CI, confidence interval; CRP, C‐reactive protein; E‐cigarette, electronic cigarette; SD, standard deviation.

Four studies assessed the effect of E‐cigarettes in comparison to cigarette smoke on dental staining (Dalrymple et al., 2018, 2021; Vohra et al., 2020; Zhao et al., 2019). Data were pooled from these, and evidence found that E‐cigarettes when compared with cigarette smoke cause less dental staining (MD: −9.137; 95% CI: −14.15, −4.58; *I* ^2^: 99%; Figure 4).

#### 3.4.2 HTPs and cigarette smoke

Five studies assessed the effects of HTPs in comparison with cigarette smoke on dental staining (Dalrymple et al., 2018, 2021; Haiduc et al., 2020; Zanetti et al., 2019; Zhao et al., 2017). We pooled the results from these and found evidence that HTPs caused less dental staining when compared with cigarette smoke (MD: −18.79; 95% CI: −23.46, −14.12; *I* ^2^: 99%; Figure 4).

#### 3.4.3 HTPs and E‐cigarette

Dalrymple et al. (2018, 2021) compared the effect of E‐cigarettes and HTPs on enamel staining. Data pooled from these studies found evidence that HTPs caused minimally more staining when compared with E‐cigarettes (MD: −0.70; 95% CI: −1.29, −0.11; *I* ^2^: 0%; Supporting Information: Figure 3).

#### 3.4.4 Types of nicotine replacement therapy

The randomized control trial by Whelton et al. (2012) assessed the effect of nicotine gum, in comparison to nicotine sublingual tablets in the reduction of stain at 6 and 12 weeks. They used a tooth‐level lobene stain index (Whelton et al., 2012) to give a staining score and a visual Vita guide score aggregate. At 6 weeks, they found evidence for the gum removed more stains than the tablet. At 12 weeks, there was no difference in stain reduction between the gum and the tablet. However, when using the Vita tooth shade assessment, they noted a small whitening effect in the gum group but not in the tablet group (Supporting Information: Table 5). This was explained by the gum preventing new stain adsorption and reducing lingual stain deposits.

Another study assessed the role of two different nicotine chewing gums in comparison to whitening chewing gum and a saliva control (Moore et al., 2008). All chewing gums had a slightly different composition; however, the nicotine chewing gums reduced staining more than the whitening gum, which in turn removed more stains than the control (Supporting Information: Table 5).

#### 3.4.5 Types of E‐cigarette flavors

One study explored the relationship between different flavors of E‐cigarettes (neutral, tobacco‐brown color, and menthol‐green color) and their resultant effect on discoloration (Pintado‐Palomino et al., 2019). They found evidence that all flavors demonstrated staining in enamel. The change in overall color when enamel was exposed to tobacco or menthol flavors was greater than neutral flavor at 0 mg/ml nicotine content (CEILab ∆E). However, according to the CIEDE200 ∆E 00 formula, they found no evidence of differences in the extent of staining between the flavors. When comparing CIELAB‐based WI_D_ values (index for assessing tooth whiteness) within the flavors, enamel exposed to menthol or tobacco scored higher values than enamel exposed to neutral flavor (Supporting Information: Table 5). In another study, Dalrymple et al. (2021) used two E‐cigarette products with the same nicotine content but different compositions and showed subtle differences between resultant color changes observed (Supporting Information: Figure 3).

#### 3.4.6 Different nicotine concentrations

Two studies assessed the relationship between varying nicotine concentrations and dental staining. Pintado‐Palomino et al. (2019) demonstrated staining in enamel above perceptibility in all concentrations of 0, 12, and 18 mg/ml of nicotine in E‐cigarette liquid. There was no evidence of dose‐dependent change linking nicotine concentration and staining. Lertsukprasert and Locharoenrat (2020) showed a change in the color of enamel above acceptability and perceptibility thresholds. They suggested a dose‐dependent increase in discoloration based on nicotine content (Supporting Information: Table 5).

*Smokeless tobacco (snus), nicotine pouches, and cigarettes*: Dalrymple et al. (2021) compared the effect of smokeless tobacco (snus), modern nicotine pouches, and cigarette smoke. Both products caused less color change than cigarette smoke (Figure 4). Nicotine pouches were seen to stain less in comparison to smokeless tobacco (Figure 2).

### 3.5 Specific components of tobacco that cause dental discoloration

One study (Haiduc et al., 2020) assessed the colored compounds within total particulate matter (TPM) deposited by cigarette smoke and HTP aerosol on enamel. After extraction with carbon disulfide, gas chromatography coupled to time‐of‐flight mass spectrometry was used to identify 11 compounds, of which 8 were terpenoids (Supporting Information: Table 7). These compounds were found in cigarette smoke TPM extract and in HTP TPM extract, suggested to be at lower levels than that of cigarette smoke TPM.

### 3.6 Comparison in levels of discoloration of dental substrates due to tobacco and nicotine products

#### 3.6.1 Enamel, dentine, and composite

Two studies assessed the differences in staining of enamel, dentine, and resin composite relative to each other (Zanetti et al., 2019; Zhao et al., 2019). One assessed staining due to exposure between cigarette smoke and E‐cigarette aerosol (Zhao et al., 2019) and the other study (Zanetti et al., 2019) compared staining with exposure between cigarette smoke and HTP smoke. They found that E‐cigarettes did cause staining in the three substrates, and this was relatively uniform. HTPs were also found to cause some staining that was similar across the three substrates. Cigarette smoke led to greater staining in comparison to both novel products. In addition, this change was also uneven amongst the substrates. Resin composite and dentine stained more than enamel. The study by Zanetti et al. (2019) suggested resin composite staining more than dentine, the other study by Zhao et al. (2019) suggested the opposite.

#### 3.6.2 Characteristics and finishing of resin composites

Eight studies compared the staining effects on different types of resin composite (Alandia‐Roman et al., 2013; Belli et al., 1997; Malhotra et al., 2011; Mathias, Costa, et al., 2010; Mathias, Silva, et al., 2010; Taraboanta et al., 2019; Theobaldo et al., 2020; Wasilewski Mde et al., 2010). The data from these studies could not be pooled due to the variation of resin composite products used and differences in methods.

When comparing the staining of different resin composite shades for staining due to cigarette smoke exposure, one study found evidence that translucent shades stained more than enamel shades within four different resin composites (Wasilewski Mde et al., 2010). None of the other studies assessed this.

Three studies assessed resin composites of different filler sizes (Alandia‐Roman et al., 2013; Malhotra et al., 2011; Theobaldo et al., 2020). Malthotra et al. (2011) found evidence that, upon exposure to smokeless tobacco solution, all three resin composites stained above the level of acceptability, but a universal micro‐hybrid composite stained less in comparison to a nanohybrid or a microhybrid posterior composite. In addition, Theobaldo et al. (2020) reported evidence that all three tested nanohybrid bulk fill composites stained above the level of acceptability and more than the microhybrid bulk fill control when exposed to cigarette smoke. Lastly, Alandia‐Roman et al. (2013) found evidence that their nanohybrid composite changed color more than a hybrid composite or a silorane‐based hybrid composite. They also compared staining in resin composite based on polishing postcuring or no polishing after the use of a smooth mylar strip. They found some evidence that the nanohybrid composite when not polished resulted in unacceptable staining but no difference between the other resin composite types (Supporting Information: Table 5). The resin composites in the studies had differing filler to resin matrix proportions with the nanohybrid and nanocomposites appearing to have a greater resin matrix component. Therefore, there was some suggestion that filler content and resin matrix proportions and properties might have some influence on how susceptible resin composites are to staining.

Mathias, Costa et al. (2010) assessed different surface finishes in resin composites and their staining from exposure to cigarette smoke (Supporting Information: Table 5). They found evidence that textured resin composite surfaces changed color more than smooth surfaces.

Belli et al. (1997) assessed direct and indirect composite and the effect of cigarette smoke exposure on their staining. They found some evidence that direct composite changed color more than indirect composite; however, this was not statistically significant (Supporting Information: Table 5).

Two studies assessed the effect of sealants on teeth and how these may change tooth color upon cigarette smoke exposure (Mathias, Silva, et al., 2010; Taraboanta et al., 2019) (Supporting Information: Table 5). Taraboanta et al. (2019) assessed the effect of three treatments used to manage white spot lesions on enamel (ICON resin infiltration, Recaldent MI Varnish, and Grandio Seal nanohybrid resin composite). They found evidence that all three products led to staining of the teeth after exposure to cigarette smoke, with ICON showing the most change. Mathias, Silva et al. (2010) assessed the placement of a surface sealant or no sealant over a nanocomposite and any resultant staining postexposure. They found evidence that both changed color above acceptability but the resin composite with a sealant on the surface stained more.

#### 3.6.3 Resin composites and dental ceramics

Two studies compared differences in staining between resin composites and dental ceramics (Belli et al., 1997; Vohra et al., 2020). The study by Belli et al. (1997) assessed the discoloration of a direct composite versus an indirect composite and a laminate ceramic to various staining agents, cigarette smoke included. They found evidence that overall direct composite stained the most, followed by indirect composite then dental ceramic. However, the effect of cigarette smoke exposure assessed in isolation at one month showed higher discoloration due to cigarette smoke in the ceramic group, not dissimilar to direct composite but statistically significantly higher than indirect composite. However, another study, (Vohra et al., 2020) comparing staining between direct composite and dental ceramic with cigarette smoke exposure, found that resin composite stained more than ceramic. This pattern was also seen when the substrates were exposed to E‐cigarette aerosol (Supporting Information: Table 5). Therefore, there was some evidence that resin composites stained more than ceramic.

#### 3.6.4 Dental ceramics and acrylic

One study assessed the difference in staining due to cigarette smoke between acrylic, high‐strength acrylic, and porcelain (Ayaz et al., 2014) and found evidence that acrylic stained more than high‐strength acrylic, which in turn stained more than porcelain (Supporting Information: Table 5).

## 4 DISCUSSION

This systematic review collated evidence from 27 studies for the four main review questions. Within the limitations of this study and the available evidence, we concluded that cigarette smoking causes staining of enamel, dentine, resin composite, dental ceramics, and acrylic. Resin composite was more susceptible to staining than enamel. E‐cigarettes were also found to cause staining, in enamel, dentine, resin composite, and ceramics, but to a lesser intensity than cigarettes and more uniformly across enamel, dentine, and resin composite. In addition, there was evidence that HTPs stained enamel, dentine, and resin composite to much lower intensity than cigarettes. However, only two studies directly compared E‐cigarettes with HTPs to suggest that HTPs stained more than E‐cigarettes. There was limited evidence that smokeless tobacco (snus) was found to cause more stains than nicotine pouches and both were found to stain less than cigarette smoke. There was limited evidence that nicotine contributed to dental staining and some nicotine‐containing gums were found to help remove stains. Finally, E‐liquid colors and flavors were shown to have an influence on discoloration.

Eleven compounds isolated from cigarette and heated tobacco particulate matter thought to be derivatives of tobacco, tobacco flavors, or pyrolysis material of plant matter were found to cause staining due to tobacco.

When comparing different resin composites for staining due to cigarette smoke, there was some evidence that nanohybrid and nanocomposites experienced more discoloration than hybrid or microhybrid composites. This was thought to be related more to the proportion of resin matrix to filler content, as opposed to filler particle size. Other factors such as surface finish, polishing postplacement, and shades of resin composite all were suggested to have some influence on the variation of resin composite staining. Finally, there was some evidence that ceramics demonstrated greater resistance to staining from cigarette smoke when compared to resin composite or acrylic.

To our knowledge, there has not been a previous systematic review completed on this subject. A mini literature review conducted by Ozsoy (2020) evaluated some of the studies (Alandia‐Roman et al., 2013; Ayaz et al., 2014; Patil et al., 2013; Raptis et al., 1982) examining the staining effects of smoking on resin composite and denture teeth. It concluded that more research was required on the color stability of different restorative materials. This present study in part supports some of the conclusions by Ozsoy (2020) but provides a more extensive review of the available evidence on tobacco and nicotine products and their effect in causing discoloration of the different dental substrates.

Little evidence is present for the exact mechanisms of how tobacco and nicotine products may discolor teeth. Scanning electron microscopy findings, by Ibrahim et al. 2019 (Ibrahim and Hassan, 2020) show that cigarette smoke exposure causes variable degrees of destruction from surface pits and holes to destruction of the rod substance of enamel. These surface irregularities could then be more susceptible to three direct staining mechanisms described by Nathoo (1997) by which chromogens could cause staining.

Published research abstracts (German et al., 2020; Holliday et al. 2020; not included in the review as full texts not available) support our findings that E‐cigarettes and heat‐not‐burn products stain resin composites less than cigarette smoke after 5 days of intense exposure. However, they also found minimal effects of E‐cigarette aerosol on resin composite unlike some studies in this systematic review. This supports our suggestion that E‐liquid compositions may have an influence on tooth staining.

This systematic review was conducted in accordance with best practices. However, some limitations may be present. The search strategy was comprehensive, but we did not include non‐English language articles, which may have biased the results. However, the three nonenglish texts we excluded were at least 30 years old. We anticipated a lack of high‐quality in vivo studies and accepted the inclusion of in vitro studies.

Most of the included studies were in vitro studies, and often had no information on sample size calculation, randomization, blinding, or details of how to access the study protocol. The studies also had varied methods, which made pooling data in meta‐analyses challenging and heterogeneity was often high (*I* ^2^ > 50%) and unexplained by our subgroups. Some of the heterogeneity can be explained by variations in products tested but accepted due to the outcome reading being that of change in color ∆E, as opposed to the exact color. This might influence the validity of the completed meta‐analysis. All these factors have a bearing on the overall quality of conclusions that can be drawn, and we would rank the evidence as low certainty.

Much of the evidence for these products comes from industry‐sponsored research into their own products. Within this systematic review, at least eight studies have declared some industry sponsorship. Whilst many were conducted to a good standard, there is still potential for industry sponsorship bias affecting the results.

From our review, it can be said with some certainty that cigarette smoking causes dental staining, which is supported by common clinical experience.

There were a small number of studies that explored the effect of novel products in comparison with cigarettes, to draw conclusions with certainty. More research is required to understand which components within E‐liquids might contribute to staining and the role of different common flavorings. These products can be modified, unlike tobacco, and if discoloring compounds are identified, an attempt could be made to eliminate these from the product.

From our results, there was some suggestion that E‐cigarettes may cause slightly less staining than HTPs. Of the 11 staining compounds isolated from cigarette smoke and HTP particulate matter extract, nicotine was not one. It has been suggested that this might be due to the extraction processes involved. The role of nicotine in causing staining is not particularly clear and studies to explore how nicotine exposure at different concentrations, compared with a nonexposure saliva control, would be beneficial to identify the role of pure nicotine in causing staining.

Validating findings with more robust evidence as to the levels of harmful or staining compounds in HTPs versus cigarette smoke is important as they are still a form of tobacco, not deemed to be risk‐free or currently accepted tools for cessation. Any advertising based on low‐quality evidence would be misleading to consumers who might seek ‘‘low‐risk’’ alternatives with a cosmetic claim. Many of the current studies are industry‐sponsored (Zanetti et al., 2019; Dalrymple et al., 2018, 2021; Zhao et al., 2017, 2019; Whelton et al., 2012) with a higher risk of bias and more independent research would be recommended.

Finally, studies with longer follow‐up times are required with variables such as daily toothbrushing and the effect of saliva controlled to give a more realistic representation of in vivo effects.

## 5 CONCLUSION

Within the limitations of this study, it can be concluded that evidence supports the clinical observation that cigarette smoke causes staining of dental hard tissues and dental materials. There was low‐quality evidence to suggest that novel tobacco and nicotine products such as E‐cigarettes and HTPs cause staining of dental hard tissues and dental materials; this was present to a lower extent than with cigarette smoke. Further independent research comparing these novel products with cigarette smoke, and each other is required. This should attempt to conduct more realistic exposures with longer‐term follow‐up. Finally, there is some suggestion that E‐liquid composition, has some influence on staining, and further research to explore this would be beneficial.

## AUTHOR CONTRIBUTIONS

**Rijula Karanjkar**: Conceptualization; investigation; writing – original draft; writing – reviewing and editing; and project administration. **Philip Preshaw**: Conceptualization; investigation; writing – review and editing; and supervision. **Janice Ellis**: Writing – review and editing; and supervision. **Richard Holliday**: Conceptualization; investigation; writing – review and editing; and supervision.

## CONFLICT OF INTEREST

The authors declare no conflict of interest.

## Supporting information

Supplementary information.

Supplementary information.

Supplementary information.

SupplementalTable 1: Completed PRISMA 2020 checklist.

Supplementary information.

Supplementary information.

Supplementary information.

Supplementary information.

Supplementary information.

Supplementary information.

## Data Availability

The data that support the findings of this study are available in the Supporting Information of this article.
