# Supplementary material for: Effect of tobacco and nicotine in causing staining of dental hard tissues and dental materials: A systematic review and meta‐analysis
Source: Clin Exp Dent Res. 2022 Nov 13;9(1):150–64. doi: 10.1002/cre2.683 (PMC9932248; doi:10.1002/cre2.683)

Supplemental Figure 1 (S1): Forest plot for sub-group analysis of discolouration of dental ceramic from exposure to cigarette smoke/extract and e-cigarette aerosol compared with a non-exposure control.


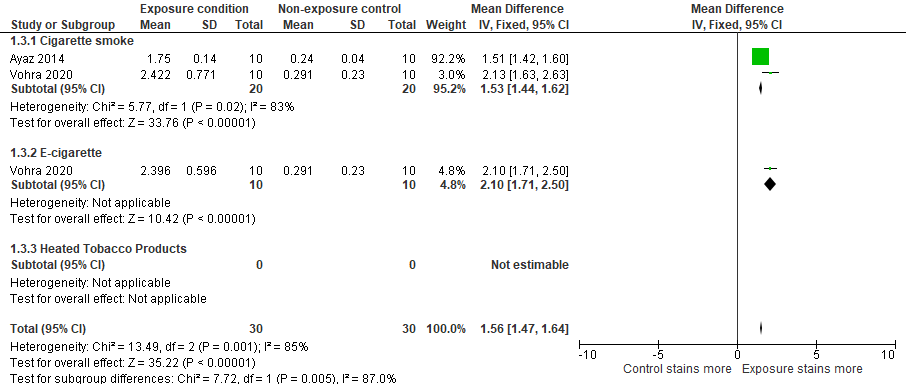

Supplement: Supplementary file 1 — Supplementary information. [file CRE2-9-150-s003.docx]
