# Supplementary material for: Effect of tobacco and nicotine in causing staining of dental hard tissues and dental materials: A systematic review and meta‐analysis
Source: Clin Exp Dent Res. 2022 Nov 13;9(1):150–64. doi: 10.1002/cre2.683 (PMC9932248; doi:10.1002/cre2.683)

Supplemental Figure 2 (S2): Forest plot for sub-group analysis of discolouration of acrylic from exposure to cigarette smoke/extract compared with a non-exposure control.


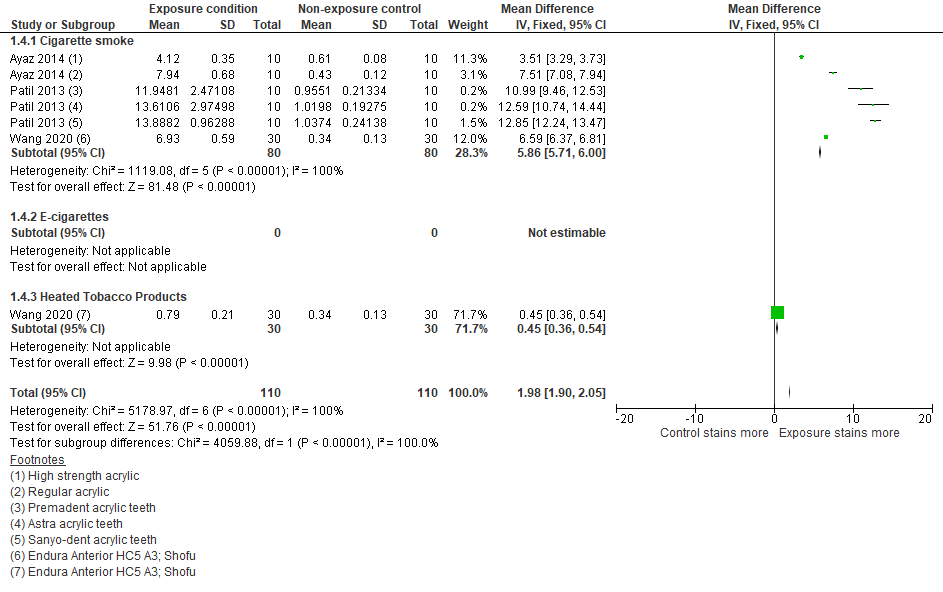

Supplement: Supplementary file 2 — Supplementary information. [file CRE2-9-150-s004.docx]
