# Supplementary material for: Effect of tobacco and nicotine in causing staining of dental hard tissues and dental materials: A systematic review and meta‐analysis
Source: Clin Exp Dent Res. 2022 Nov 13;9(1):150–64. doi: 10.1002/cre2.683 (PMC9932248; doi:10.1002/cre2.683)

Supplemental Figure 3 (S3): Forest plot of sub-group analysis for comparison of e-cigarette and heated tobacco product aerosol/ extract exposure on enamel substrate.


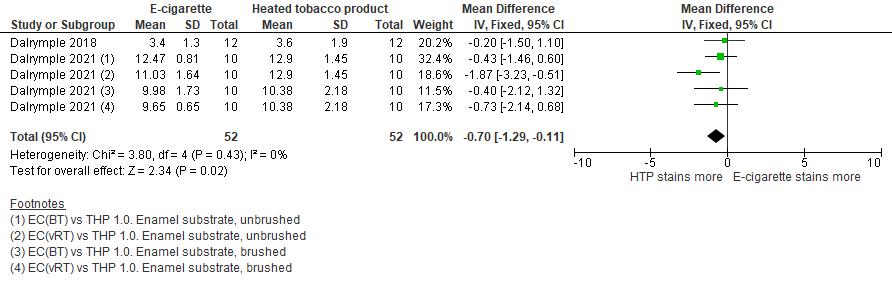

Supplement: Supplementary file 3 — Supplementary information. [file CRE2-9-150-s001.docx]
