# Supplementary material for: Effect of tobacco and nicotine in causing staining of dental hard tissues and dental materials: A systematic review and meta‐analysis
Source: Clin Exp Dent Res. 2022 Nov 13;9(1):150–64. doi: 10.1002/cre2.683 (PMC9932248; doi:10.1002/cre2.683)
Supplement: Supplementary file 5 — Supplementary information. [file CRE2-9-150-s005.docx]

Supplemental table 2: Search strategy

|  | Concept #1 | Concept #2 | Concept #3 |
| --- | --- | --- | --- |
| text words | tooth$  enamel$  dentin$  composite$  acrylic$  porcelain$  resin composite$  ceramic$  amalgam$  dental materials$  dental$  denture$ | stain$  discolour$  discolor$  pigment$  extrinsic$  intrinsic$ | nicotine (MeSH)  nicotine$  tobacco (MeSH)  tobacco$  electronic nicotine delivery system  vaping (MeSH)  vap$  electronic cigarette$  E-cigarette$  heat-not-burn$  heated tobacco$  shisha$  smokeless tobacco (MeSH)  smokeless tobacco$  chewing tobacco$  nicotine chewing gum  nicotine lozenges  nicotine oral strips  nicotine mouth spray  nicotine nasal spray  nicotine replacement therapy  pan  betel quid  snus  snuff |
