# Supplementary material for: Effect of tobacco and nicotine in causing staining of dental hard tissues and dental materials: A systematic review and meta‐analysis
Source: Clin Exp Dent Res. 2022 Nov 13;9(1):150–64. doi: 10.1002/cre2.683 (PMC9932248; doi:10.1002/cre2.683)
Supplement: Supplementary file 6 — Supplementary information. [file CRE2-9-150-s007.pdf]

Title:	
Author:	
Year:	
Journal:	
Abstract and full text language :	
Location of first author:	
Funding:	
Type of study:	RCT<br>Non-randomised clinical study<br>(cohort studies, case-control studies, cross-sectional studies)<br>Clinical survey/ epidemiological study<br>In vitro laboratory study
Form of tobacco/nicotine:	
Substrate studied:	Tooth<br>Enamel<br>Dentine<br>Resin composite<br>Porcelain<br>Silicone<br>Acrylic<br>Other:
Suitable control with no smoke exposure	Yes/No
Does the study investigate the effect of tobacco/smoke on discoloration?	Yes/No<br>If so, what are the results?
Does the study look at specific components of tobacco/smoke?	Yes/No<br>If so, what are the results?
Does the study investigate the effect of nicotine alone on discoloration?	Yes/No
Does the study compare different tobacco and nicotine products?	Yes/No<br>If so, what are the results?
Include/exclude	If excluded, reasons:

Supplemental table 3: Data collection sheet template
