# Supplementary material for: Effect of tobacco and nicotine in causing staining of dental hard tissues and dental materials: A systematic review and meta‐analysis
Source: Clin Exp Dent Res. 2022 Nov 13;9(1):150–64. doi: 10.1002/cre2.683 (PMC9932248; doi:10.1002/cre2.683)
Supplement: Supplementary file 7 — Supplementary information. [file CRE2-9-150-s002.docx]

Supplemental table 4: Reasons for exclusion

| **No** | **Study author** | **Year** | **Exclusion reason** |
| --- | --- | --- | --- |
| 1 | Addy | 1979 | No non-tobacco/nicotine exposure control |
| 2 | Al-Shammari | 2005 | Survey reporting patient perceptions no investigation into effect of `tobacco/ nicotine on dental stain |
| 3 | Andersson | 2016 | Patient reported survey. No non-exposure control data |
| 4 | Barbosa | 2018 | Lack of clarity in results, not enough data presented. |
| 5 | Bazzi | 2012 | No non-exposure control |
| 6 | Bertoldo | 2011 | No non-exposure control |
| 7 | Christen | 1985 | Methodological issues, unclear if baseline colour readings accurate and relevant |
| 8 | Da Silva | 2017 | Effect on fluorescence measured not colour change |
| 9 | De gues | 2018 | No non-exposure control |
| 10 | Eswar | 2002 | No non-exposure control; confounding variables not controlled |
| 11 | Hassab | 2021 | No details of tobacco exposure provided therefore not able to establish any relationships. |
| 12 | Imirzalioglu | 2010 | Methodology not clear, whether nicotine or tobacco solution used |
| 13 | Indrapriyadarshini | 2019 | No non-exposure control |
| 14 | Knychalska-Karwan Z | 1968 | Full text unable to translate, not found |
| 15 | Luce | 1988 | Old study, material not used in current practise due to advances |
| 16 | Mallikarjuna | 2012 | Case series. No non-exposure control |
| 17 | Mathais | 2014 | Effect on solubility and sorption measured not colour change |
| 18 | Nasir | 2017 | No non-exposure control; confounding variables not controlled |
| 19 | Okur | 2004 | Full text unable to translate. Abstract reviewed in English no-non exposure control |
| 20 | Prayitno | 1979 | Confounding variables not controlled |
| 21 | Publio | 2013 | No non-exposure control |
| 22 | Raptis | 1982 | Old study, material not used in current practise due to advances |
| 23 | Robertson | 1997 | Review of studies, unclear how data combined |
| 24 | Roulet-Mehrens | 1982 | Old study, material not used in current practise due to advances |
| 25 | Satou | 1984 | Full text unable to translate |
| 26 | Schmitd | 1952 | Full text unable to translate |
| 27 | Shintani | 1984 | Old study, material not used in current practise due to advances |
| 28 | Shintani | 1985 | Old study, material not used in current practise due to advances |
| 29 | Yu | 1983 | No non-exposure control |
