# Supplementary material for: Effect of tobacco and nicotine in causing staining of dental hard tissues and dental materials: A systematic review and meta‐analysis
Source: Clin Exp Dent Res. 2022 Nov 13;9(1):150–64. doi: 10.1002/cre2.683 (PMC9932248; doi:10.1002/cre2.683)
Supplement: Supplementary file 8 — Supplementary information. [file CRE2-9-150-s006.docx]

Supplemental table 5: Included study characteristics (Brief data collection notes)

| **Alandia-Roman 2012** |  |
| --- | --- |
| Method | Study design: *In vitro* lab study  Location: Brazil |
| Population | Substrates:  resin composites: Filtek Z 250; Tetric N- Ceram; Filtek P90  n: 20 per composite  Split half into polished and unpolished |
| Intervention | Exposure type: cigarette smoke Marlboro Red (Philip Morris)  Exposure duration: 10 minutes each cigarette; 20 cigarettes per sample brushed in a standardized manner after each cigarette exposure |
| Control | Baseline/ composite control |
| Outcome | Colour change measurement: ∆E CIELAB  Longest exposure: 20 cigarettes  No time given  Data: Means for ∆E (standard deviation)  Tetric N-Ceram: polished 3.08 (0.56); unpolished 3.39 (0.63)  Z 250: Polished 1.87 (0.48); unpolished 2.11 (0.53)  P 90: Polished 1.79 (0.44); unpolished 2.02 (0.48)  Tetric compared to the other 2 composites reach statistical difference |
| Funding source | No funding declared |
| Author conflict of interest | Declared no conflict of interest |
| Notes |  |
| **Alkhatib 2005** |  |
| Method | Study design: Epidemiological study  Location: UK |
| Population | Smokers 817 |
| Intervention | Exposure type: smoking |
| Control | Non-smokers 2567 |
| Outcome | Colour change measurement: self-reported discolouration based on comparison to photographs of varying stain levels.  Logistic regression of smoking and tooth discolouration:  Mild discolouration odds ratio 0.94 non-significant  Moderate discolouration odds ratio 1.76 statistically significant  Severe discolouration odds ratio 2.43 statistically significant  Reference is non-smokers. |
| Funding source | United Kingdom Department of Public Health |
| Author conflict of interest | UK declared no conflict of interest |
| Notes |  |
| **Amorim 2020** |  |
| Method | Study design: *In vitro* lab study  Location: Brazil |
| Population | Substrates: Bovine teeth  n: 6 |
| Intervention | Exposure type: Cigarette smoke  Exposure duration:  Samples stored in human saliva for 2 hours a day for 15 days  5 cycles of 4 cigarettes (20 in total)  Samples brushed with a standardized technique after each exposure cycle |
| Control | Distilled water 37 $℃$ 30 days  n= 6 |
| Outcome | Colour change measurement: (CIEDE2000)  Longest exposure: 15 days |
| Funding source | Brazilian government agency: CAPES |
| Author conflict of interest | No conflict declared |
| Notes | Data for analysis:  To estimate $\Delta E$ from graph but E 00 not E0 (different formula) so not suitable for meta-analysis. Also, challenging to read scale from graph due to small spacing. |
| **Ayaz 2014** |  |
| Method | Study design: *In vitro* lab study  Location: Turkey |
| Population | Substrates: Acrylic: Polymethyl methacrylate; Reinforced polymethyl methacrylate (high strength acrylic) denture teeth  Porcelain denture teeth  n: 10 |
| Intervention | Exposure type: Cigarette Smoke  Exposure duration: 20 Cigarettes, 10 mins each  Washed with running water after exposure. |
| Control | n=10 distilled water  Means (Standard deviation):  Acrylic resin: 0.43 (0.12)  High strength acrylic: 0.61 (0.08)  Porcelain: 0.24 (0.04) |
| Outcome | Colour change measurement: ∆E CIELAB  Longest exposure: 20 cigarettes  Mean ∆E (standard deviation):  Acrylic resin: 7.94 (0.68)  High strength acrylic: 4.12 (0.35)  Porcelain: 1.75 (0.14)  Statistically significant difference between all three materials |
| Funding source | Not noted |
| Author conflict of interest | Not declared |
| Notes | Data for meta-analysis: As above |
| **Belli 1997** |  |
| Method | Study design: *In vitro* lab study  Location: Turkey |
| Population | Substrates: Composite resin- Direct hybrid 3M Velux plus; Indirect EOS inlay microfilled.  Ceramic- Ceramco II laminating porcelain  n: 6 in each arm |
| Intervention | Exposure type: Cigarette smoke  Exposure duration: 300 cigarettes /1 month |
| Control | Distilled water control n=6 |
| Outcome | Colour change measurement: ∆E CIELAB mean  Longest exposure: 1 month  Statistically significant difference between control and all three materials  Direct composite: 6.71  Indirect composite: 5.87  Porcelain: 7.80  No standard deviation given |
| Funding source | Not stated |
| Author conflict of interest | Not stated |
| Notes | Data: estimation from graphs. |
| **Dalrymple 2018** |  |
| Method | Study design: *In vitro* lab study  Location: UK |
| Population | Substrates: Bovine enamel blocks  n: 10 exposures to particulate matter; 12 exposures to whole smoke |
| Intervention | Exposure type: 3R4F cigarette smoke, Heated Tobacco Product THP 1.0 device with Neostick, E-cigarette NVP Twilight Tobacco  Exposure to particulate matter in one study and to whole aerosol in another study.  Exposure duration: 14 days/200 puffs x 5 days respectively |
| Control | DMSO/ Phosphate buffered saliva n= 10/12  Mean (Standard deviation)  Control DMSO: 12.6 (2.0)  Control phosphate buffered saline: 5.3 (0.8) |
| Outcome | Colour change measurement: ∆E CIELAB  Longest exposure: 14 days/ 5 days then colour change also noted at 30 days  Mean ∆E (Standard deviation):  3R4F 29.4 (3.6)  THP 1.0 10.5 (2.3)  NVP 10.7 (2.6)  Exposure to whole smoke aerosol:  3R4F 26.2 (3.2)  THP 1.0 3.6 (1.9)  NVP 3.4 (1.3) |
| Funding source | British American Tobacco |
| Author conflict of interest | Authors have declared they are employees of BAT, Intertek CRS or Borgwaldt KC GmbH |
| Notes | Data for meta-analysis: As above |
| **Dalrymple 2021** |  |
| Method | Study design: *In vitro* lab study  Location: UK |
| Population | Substrates: Bovine enamel  n: 20. 10 brushed 10 not brushed |
| Intervention | Exposure type: 1R6F cigarettes; THP 1.0 heated tobacco product; E-cigarettes with blended tobacco (BT); rich tobacco e-liquid (vRT); CAS extracted reference snus (CRP 1.1); modern oral product (LYFT)  DMSO solvent used to extract product to stain |
| Control | Negative control artificial saliva and DSMO solvent.  Mean ∆E (standard deviation):  Artificial saliva 1.12 (0.75)  DMSO solvent 12.21 (1.88) |
| Outcome | Colour change measurement: ∆E CIELAB  Longest exposure: 64 days |
| Funding source | British American Tobacco |
| Author conflict of interest | Declared |
| Notes | Data for meta-analysis: Day 14 mean ∆E and standard deviation  Cigarette 20.41(2.18)  HTP 12.90 (1.45)  EC (BT) 12.47 (0.81)  EC (vRT) 11.03 (1.64)  Snus 10.39 (2.92)  LYFT 2.22 (1.53) |
| **Haiduc 2020** |  |
| Method | Study design: *In vitro* lab study  Location: Switzerland |
| Population | Substrates: bovine enamel  n: 20 |
| Intervention | Exposure type: Total particulate matter (TPM) from 3R4F cigarette smoke or heated tobacco product THS 2.2 aerosol in solution with artificial saliva.  Exposure duration: 14 days  Brushing of samples in standardized manner at 7 and 14 days |
| Control | n= 20 in Artificial saliva  Mean ∆E (standard deviation)  control 0.68 (0.38) |
| Outcome | Colour change measurement: (CIELAB)  Longest exposure: 14 days, interim measurement at 7 days  Identification of compounds in cigarette smoke (CS) and THS total particulate matter extracted from enamel: 11 compounds isolated as main contributors to staining.  14 days, post brushing mean ∆E and standard deviation.  Cigarette smoke TPM 19.6 (8.14)  THS TPM 1.96 (0.51)  Difference between control and cigarette smoke TPM is statistically significant. Difference between control and THS 2.2 TPM is not statistically significant. |
| Funding source | Philip Morris international |
| Author conflict of interest | Authors declare some of them are employees and paid by Philip Morris international |
| Notes | Data for meta-analysis: As above. |
| **Kobayashi 2021** |  |
| Method | Study design: *In vitro* lab study  Location: Brazil |
| Population | Substrates: Bovine enamel blocks  n: 10 |
| Intervention | Exposure type: Cigarette smoke  Exposure duration: 20 cigarettes for 5 days |
| Control | Artificial saliva control |
| Outcome | Colour change measurement: $\Delta E$CIELAB  Longest exposure: 20 cigarettes a day/ 5 days |
| Funding source | CAPES Brazilian government funding |
| Author conflict of interest | None declared |
| Notes | Data for analysis: Could not estimate exact values from graph but calculation from graph values in $\Delta E$CIELAB shows change in colour from cigarette smoke about twice that of control (mean 12.02 vs 6.45). Contacted author for results via email but no response. |
| **Lertsukprasert 2020** |  |
| Method | Study design: *In vitro* lab study  Location: Bangkok |
| Population | Substrates: Enamel model  n: 9 in each nicotine concentration |
| Intervention | Exposure type: Immersion of calcium phosphate enamel model in nicotine solution of 6,11,16,21 mg/ml for 15 mins  Exposure duration:15 mins |
| Control | Compared to baseline colour change from 0 mg/ml nicotine solution |
| Outcome | Colour change measurement: ∆E CIELAB  Longest exposure: 15 mins  Nicotine Concentration (mg/ml) and the mean ∆E:  6: 4.18  11: 4.43  16: 4.69  21: 4.97  No error margins for these readings |
| Funding source | Funding from Kind Mongkut Institute of Technology |
| Author conflict of interest | Declared no conflict of interest |
| Notes | Data for meta-analysis: Not suitable |

| **Malhotra 2011** |  |
| --- | --- |
| Method | Study design: *in vitro* lab study  Location: India |
| Population | Substrates: Resin composites  Microhybrid posterior P60  Universal microhybrid Z100  Nanocomposite Ceram-X mono  n: 12 |
| Intervention | Exposure type: chewing tobacco solution  Exposure duration: 3 hours/ day, 15 days |
| Control | Baseline/ material comparison |
| Outcome | Colour change measurement: $\Delta E$CIELAB  Longest exposure: 15 days  Mean $\Delta E$ (standard deviation)  P60: 18.83 (2.29) a  Z100: 13.59 (1.37) ab  Ceram-X-mono: 18.83 (3.07) b  Same letter indicates statistically significant difference |
| Funding source | Dentsply supported this project with supply of Ceram- X mono |
| Author conflict of interest | Declared no financial interest |
| Notes |  |
| **Mathias 2010** |  |
| Method | Study design: *In vitro* lab study  Location: Brazil |
| Population | Substrates: Resin composite (Filtek supreme XT)  Smooth surface and texturized  n: 10 per arm |
| Intervention | Exposure type: Cigarettes  Exposure duration: 21 days, daily 20 cigarettes |
| Control | Non exposure control  smooth surface n=10, 21 days  texturized surface n=10, 21 days  Stored in artificial saliva  Non exposure control: mean $\Delta E$(standard deviation)  smooth surface 8.2 (0.6)  texturized surface 6.3 (0.4) |
| Outcome | Colour change measurement: $\Delta E$CIELAB  Longest exposure: 21 days  Mean $\Delta E$(standard deviation)  Cigarettes:  Smooth surface stained 12.1 (1.7)  Texturized surface stained 16.1 (0.9) |
| Funding source | Products donated by 3M ESPE and KG Sorenson |
| Author conflict of interest | Authors have declared no financial interest |
| Notes |  |

| **Mathias 2010** |  |
| --- | --- |
| Method | Study design: *In vitro* lab study  Location: Brazil |
| Population | Substrates: Resin composite nanocomposite (Filtek supreme XT) with or without surface sealant (Bioforty)  n: 10 |
| Intervention | Exposure type: Cigarette smoke  Exposure duration: 10 cigarettes 8 mins, twice daily for 21 days |
| Control | Baseline/ comparison between material finishes |
| Outcome | Colour change measurement: $\Delta E$CIELAB  Longest exposure: 20 cigarettes a day/ 21 days  Mean $\Delta E$(standard deviation) between baseline and staining  No sealant: 12.07 (1.73)  Sealant: 18.45 (3.09)  Statistically significant difference between sealant and no-sealant. Sealant stains worse. |
| Funding source | None declared |
| Author conflict of interest | None declared |
| Notes |  |
| **Mathias 2011** |  |
| Method | Study design: *In vitro* lab study  Location: Brazil |
| Population | Substrates: Resin composite (Filtek Z350)  n: 10 in each arm |
| Intervention | Exposure type: Cigarette smoke (CS) (Hollywood Red KA Souza cruz S.A)  Exposure duration: 20 cigarettes daily (10 over 8 minutes twice daily) for 21 days  n= 10 |
| Control | Non-colourant control stored in artificial saliva  Mean ∆E (standard deviation)  8.19 (0.63) |
| Outcome | Colour change measurement: ∆E CIELAB  Longest exposure: 21 days  Perceptible change of colour between cigarette smoke and control but no statistically significant difference between cigarette smoke and control |
| Funding source | None declared |
| Author conflict of interest | None declared |
| Notes | Data for meta-analysis: Colour change at 21 days  Cigarette smoke mean ∆E (standard deviation): 12.07 (1.73) |
| **Moore 2008** |  |
| Method | Study design: *In vitro* lab study  Location: USA |
| Population | Substrates: Bovine enamel  n:16 |
| Intervention | Exposure type: Nicotine chewing gum (A and B) both 2 mg nicotine, difference in Calcium carbonate content  Exposure duration: 120 mins to chewing stimulation with human saliva |
| Control | Gum C is a positive control- confectionary whitening gum in addition to a saliva only control.  Mean ∆E (standard deviation):  Gum C (control): 6.55 (2.44)  Saliva: 0.65 (0.29)  Statistically significant difference between Gum C and saliva, with saliva alone removing least stain |
| Outcome | Colour change measurement: ∆E CIELAB  Longest exposure: 120 mins  Mean ∆E (standard deviation):  Gum A: 14.91 (2.12)  Gum B: 13.15 (1.57)  No significant difference between Gum A and B  Statistically significant difference between gum A/B vs gum C and saliva |
| Funding source | Novartis consumer health |
| Author conflict of interest | 2nd and 3rd author are employees of Novartis; decaled no conflict of interest |
| Notes | Data for analysis:  Nicotine gum not replaced but whitening gum replaced every 20 mins. Reduction in stain measured. Different composition of gums especially nicotine replacement therapy (NRT) vs confectionary. Gum chewing effect on removal of stain outweighs any potential staining through nicotine and gum properties of NRT may lead to it being more abrasive in this study vs the positive control |
| **Ness 1977** |  |
| Method | Study design: Epidemiological study  Location: UK |
| Population | Substrates: dental patients who smoke  n= 1176 |
| Intervention | Exposure type: Use of tobacco in cigarettes, pipes or cigars |
| Control | Dental patients who are non-smoker n= 3128 |
| Outcome | Colour change measurement: Dentist recorded degree of staining as mild, moderate and severe.  Patient reported data on use of tobacco.  Total sample 27% smokers vs nonsmokers  50 % of smokers experience moderate to severe smoking vs 16% of non- smokers |
| Funding source | Employee of Proctor & Gamble |
| Author conflict of interest | None declared |
| Notes |  |
| **Patil 2013** |  |
| Method | Study design: *In vitro* lab study  Location: India |
| Population | Substrates: Acrylic types (A/B/C)  n: 10 |
| Intervention | Exposure type: Cigarette smoke  Exposure duration: 6 cigarettes daily, 9 puffs each cigarette, lasting 2 s, 1 puff every 60 s for 21 days |
| Control | Artificial saliva control n=10  Mean ∆E (standard deviation):  acrylic A: 0.96 (0.21)  acrylic B: 1.02 (0.19)  acrylic C: 1.04 (0.24) |
| Outcome | Colour change measurement: ∆E CIELAB  Longest exposure: 21 days  Mean ∆E (standard deviation):  Cigarette smoke exposure:  acrylic A: 11.9481 (2.47108)  acrylic B: 13.6106 (2.97498)  acrylic C: 13.8882 (0.96288) |
| Funding source | None |
| Author conflict of interest | Declared no conflicts of interest |
| Notes | Data for meta-analysis: Outcome data |
| **Pintado- Palomino 2018** |  |
| Method | Study design: I*n vitro* lab study  Location: Brazil |
| Population | Substrates: Bovine enamel  n: 7 |
| Intervention | Exposure type: Different e-liquid flavors (neutral, menthol, tobacco) and different nicotine content (12 and 18 mg)  Exposure duration: 20 cycles (200 puffs) |
| Control | Baseline and 0 mg nicotine control |
| Outcome | Colour change measurement: ∆E CIELAB  Longest exposure: 200 puffs  Mean ∆E (standard deviation):   \| mg \| Neutral \| Menthol \| Tobacco \| \| --- \| --- \| --- \| --- \| \| 0 \| 2.40 (1.10) aA \| 4.60 (1.80) aB \| 3.10 (1.30) aAB \| \| 12 \| 1.90 (1.40) aA \| 2.40 (1.70) bA \| 3.60 (1.90) aA \| \| 18 \| 3.60 (2.10) aA \| 3.10 (1.60) abA \| 3.20 (1.80) aA \|   For each formula, different letters, lowercase in columns, and uppercase in rows indicate statistically significant difference. |
| Funding source | CAPES- Government funding |
| Author conflict of interest | Authors have declared no conflict of interest |
| Notes | Data for meta-analysis: as above  Visually perceptible colour change in all flavors and contents. Mixed results for acceptability but mostly all presented with change above unacceptability threshold. No statistically significant interaction between flavor and nicotine content. (p= 0.17) |
| **Taraboanta 2019** |  |
| Method | Study design: *In vitro* lab study  Location: Romania |
| Population | Substrates: Human enamel from teeth with demineralized white spot lesions created which were then treated with ICON infiltration resin therapy; remineralization Recaldent MI varnish; sealing with Grandio seal nanohybrid composite resin.  n: 10 |
| Intervention | Exposure type: Cigarette smoke  Exposure duration: 8 min/ cycle 20 times a day for 21 days |
| Control | n=10 stored in artificial saliva  Control results:  Mean ∆E (standard deviation):  ICON: 2.4 (1.6)  Recaldent MI-varnish: 2.6 (1.9)  Grandio seal: 2.3 (1.7) |
| Outcome | Colour change measurement: ∆E CIELAB  Longest exposure: 21 days  Mean ∆E (standard deviation):  ICON: 34.5 (3.9)  Recaldent MI-varnish: 17.7 (2.3)  Grandio seal: 24.3 (3.7) |
| Funding source | Not reported |
| Author conflict of interest | Not reported |
| Notes | Data for meta-analysis: see above |
| **Theobaldo 2020** |  |
| Method | Study design: *In vitro* lab study  Location: Brazil |
| Population | Substrates: Different high viscosity bulk-fill composites  n:10 |
| Intervention | Exposure type: Cigarette smoke (CS) Marlboro red 10 pack  Exposure duration: 1 x 10 pack |
| Control | Baseline. Z 250 XT composite |
| Outcome | Colour change measurement: ∆E CIELAB  Longest exposure: 1x10 pack  Mean ∆E (standard deviation):  Filtek Z250 XT 2.0 (0.4) c  Filtek one bulk- fill 3.8 (1.2) b  Tetric N-Ceram Bulk-fill 4.4 (0.7) ab  Aura Bulk-fill 5.6 (1.3) a  Different lowercase letter indicates statistical differences |
| Funding source | CAPES Brazilian government agency. |
| Author conflict of interest | Declared no conflict of interest |
| Notes |  |
| **Vohra 2020** |  |
| Method | Study design: *In vitro* lab study  Location: Saudi Arabia |
| Population | Substrates: Resin composite (Core Restore2- Kerr)  Ceramic (Empress- Ivoclar vivadent)  n: 10 in each exposure arm. |
| Intervention | Exposure type: Cigarette smoke (CS) (Marlboro gold – PM)  E-cigarette (EC) (SMOK- Alien, Cloudniners Mango Nicotine strength 3 mg)  Exposure duration: 2s per puff, 10 puffs per cycle, 10 cycles per day for 7 days |
| Control | No smoke control. n=10 stored in artificial saliva  Mean $\Delta E$ (Standard deviation):  Ceramic: 0.291(0.23)  Resin composite: 0.558 (0.329) |
| Outcome | Colour change measurement: $\Delta E$CIELAB  Longest exposure: 7 days  Mean $\Delta E$ (Standard deviation):  Ceramic:  CS 2.422 (0.771) statistically significant difference compared with control  EC 2.396 (0.596) statistically significant difference compared with control  Resin composite:  CS 42.871 (2.448) statistically significant difference compared with control  EC 46.866 (3.641) statistically significant difference compared with control  No Statistical significance when compared EC with CS |
| Funding source | King Saud University project number (RSP- 2019-44) |
| Author conflict of interest | Declared no conflict of interest |
| Notes | Data for meta-analysis: as above |
| **Wang 2020** |  |
| Method | Study design: *In vitro* lab study  Location: Brazil |
| Population | Substrates: Acrylic teeth  n: 30 each |
| Intervention | Exposure type: Marlboro medium cigarette smoke/ IQOS 2.4 THP smoke (Heated tobacco product)  Exposure duration: 15 cigs 7 days in total |
| Control | Air  7 days mean (standard deviation):  Control air 0.34 (0.13) |
| Outcome | Colour change measurement: ∆E CIELAB  Longest exposure: 15 cigs/ 7 days  7 days mean (standard deviation):  Cigarette smoke 6.93 (0.59)  Heated tobacco product smoke 0.79 (0.21)  Statistically significant difference between control and both exposures. |
| Funding source | Research fund |
| Author conflict of interest | None declared |
| Notes | Data for analysis: as above |
| **Wasilewski 2010** |  |
| Method | Study design: *In vitro* lab study  Location: Brazil |
| Population | Substrates: Resin composites enamel and translucent shades of 5 types: Grandio; Charisma; Filtek Supreme XT; Opalis; 4 seasons  n: 10 per shade |
| Intervention | Exposure type: Cigarette smoke Marlboro  Exposure duration: 5 cigarettes x 4 cycles |
| Control | Baseline/composite comparison |
| Outcome | Colour change measurement: ∆E CIELAB  Longest exposure: 20 cigarettes  Mean ∆E (standard deviation):  Grandio: Enamel 9.4 (0.6); Translucent 16.9 (1.6)  Filtek Supreme XT: Enamel 9.7 (0.3); Translucent 10.0 (1.2)  Charisma: Enamel 10.2 (1.1); Translucent 18.1 (0.8)  Opallis: Enamel 9.0 (0.9); Translucent 17.2 (3.8)  4 Seasons: Enamel 7.0 (0.8); Translucent 12.5 (1.7) |
| Funding source | Material supply from 3M ESPE, FGM Dental products, Heraeus Kulzer GmbH & Co and VOCO GmbH |
| Author conflict of interest | Declared no conflict of interest |
| Notes |  |
| **Whelton 2012** |  |
| Method | Study design: RCT  Location: Ireland |
| Population | Substrates: Smokers’ teeth  n: 200 (102 gum/ 98 tablet) |
| Intervention | Exposure type: Nicotine gum vs Nicotine Tablet  Exposure duration: 12 weeks |
| Control | Comparison between gum and microtab |
| Outcome | Colour change measurement: Lobene stain index and Vita shade guide  Primary outcome was mean change in stain score at 6 weeks. Secondary outcome was Vita shade guide change between baseline and 2,6 and 12 weeks.  Longest exposure: 12 weeks  At 12 weeks no statistically significant difference in between tablet and gum for change in stain. Change in Vita shade assessment with the gum being statistically significantly lighter than tablet. |
| Funding source | Study funded by AB McNeil who is the manufacturer of the products used in the study, externally monitored by MDS Pharma |
| Author conflict of interest | Competing interests declared by one author |
| Notes | Not included in meta-analysis |
| **Zenetti 2019** |  |
| Method | Study design: *In vitro* lab study  Location: NY/USA |
| Population | Substrates: Human enamel, dentine and resin composite.  n: 11 |
| Intervention | Exposure type: 3R4F cigarette smoke (CS) and THS 2.2 aerosol (heated tobacco product)  20 cigs/20 sticks a day  Exposure duration: 12 days over 3 weeks (Mon-Thurs)  Brushing at the end of each week in a standardized manner |
| Control | Baseline/ compare with each other |
| Outcome | Colour change measurement: ∆E CIELAB  Longest exposure: 12 days (3 weeks)  Week 3 after brushing mean ∆E (standard deviation):  Enamel: 3R4F 8.8 (2.6); THS 2.8 (1.2)  Dentine: 3R4F 21.3 (4.4); THS 3.1 (0.8)  Composite resin: 3R4F 25.6 (3.8); THS 3.0 (1.0) |
| Funding source | In part funded by Philip Morris international |
| Author conflict of interest | Some authors declared they are employees of Philip Morris International |
| Notes | Data for meta-analysis: as above |
| **Zhao 2017** |  |
| Method | Study design: *In vitro* lab study  Location: USA |
| Population | Substrates: Resin composite types (DVS, TEC, FSU)  n:10 aged and 10 non- aged |
| Intervention | Exposure type: 3R4F cigarettes/ THS 2.2 (heated tobacco product)  Exposure duration: 20 sticks/cigs a day 12 days over. 3 weeks |
| Control | Baseline/ comparison of the 2 exposures |
| Outcome | Colour change measurement: ∆E CIELAB  Longest exposure: 12 days/ 3 weeks  Mean ∆E (standard deviation):  DVS: 3R4F 23.0 (1.2); THS 4.0 (0.6) statistically significant difference comparing cigarette smoke to HTP  TEC: 3R4F 30.4 (1.4); THS 5.3 (1.5) statistically significant difference comparing cigarette smoke to HTP  FSU: 3R4F 28.0 (2.5); 2.6 (0.5) statistically significant difference comparing cigarette smoke to HTP |
| Funding source | Philip Morris International |
| Author conflict of interest | Authors have declared they are employees of Philip Morris International |
| Notes | Data for meta-analysis: see above |
| **Zhao 2019** |  |
| Method | Study design: *In vitro* lab study  Location: NY, USA |
| Population | Substrates: Human Enamel, Dentine, Resin composite  n: 20 |
| Intervention | Exposure type:  3R4F cigarettes smoke (CS) 56 mins 20 cigarettes a day  E-cigarette (EC) MESH classic tobacco 300 puffs a day  Exposure duration: 15 days  Brushing in standardized manner every day |
| Control | Baseline/ compare with each other |
| Outcome | Colour change measurement: ∆E CIELAB  Longest exposure: 15 days, results post brushing  Mean ∆E (standard deviation):  Enamel: CS 8.37 (2.33); EC 2.27 (0.53) Statistically significant difference comparing CS and EC  Dentine: CS 21.44 (4.44); EC 2.81 (0.91) Statistically significant difference comparing CS and EC  Composite resin: CS 13.73 (4.18); EC 2.37 (1.00) Statistically significant difference comparing CS and EC |
| Funding source | In part by Philip Morris International |
| Author conflict of interest | Some authors declared they are employees of Philip Morris international |
| Notes | Data for meta-analysis: as above |

CIELAB- International Commission on Illumination colour space

CIEDE2000- colour difference equation

∆E – change in colour

n - number

CAPES- Coordination for the improvement of higher education personnel

DMSO-Dimethyl sulfoxide solvent

3R4F- reference cigarette

CS- Cigarette smoke

EC- E-cigarette

HTP- Heated tobacco product

TPM- Total particulate matter
