# Supplementary material for: Effect of tobacco and nicotine in causing staining of dental hard tissues and dental materials: A systematic review and meta‐analysis
Source: Clin Exp Dent Res. 2022 Nov 13;9(1):150–64. doi: 10.1002/cre2.683 (PMC9932248; doi:10.1002/cre2.683)
Supplement: Supplementary file 9 — Supplementary information. [file CRE2-9-150-s008.docx]

Supplemental table 6: Reporting quality assessment for studies.

a. In Vitro studies

| **Reporting quality Assessment modified CONSORT checklist- In vitro studies** | | | | |  |  |  |  |  |  |  |  |
| --- | --- | --- | --- | --- | --- | --- | --- | --- | --- | --- | --- | --- |
| ITEM | Amorim 2020 | Wasilewski 2010 | Mathias 2010 | Lertsukprasert 2020 | Mathias 2011 | Moore 2008 | Belli 1997 | Ayaz 2014 | Malhotra 2011 | Alandia -Roman 2012 | Dalrymple  2021 | Haiduc 2020 |
| 1 | Yes | yes | yes | yes | yes | yes | yes | yes | yes | yes | yes | yes |
| 2a | yes | yes | yes | yes | yes | yes | yes | yes | yes | yes | yes | yes |
| 2b | no | yes | yes | yes | yes | yes | no | yes | yes | yes | yes | yes |
| 3 | yes | yes | yes | yes | yes | yes | yes | yes | yes | yes | yes | yes |
| 4 | yes | yes | yes | yes | yes | yes | yes | yes | yes | yes | yes | yes |
| 5 | no | no | no | no | no | no | no | no | no | no | no | no |
| 6 | no | no | no | no | no | no | no | no | no | no | no | no |
| 7 | no | no | no | no | no | no | no | no | no | no | no | no |
| 8 | no | no | no | no | no | no | no | no | no | no | no | no |
| 9 | no | no | no | no | no | no | no | no | no | no | no | no |
| 10 | yes | yes | yes | yes | yes | yes | yes | yes | yes | yes | yes | yes |
| 11 | No | yes | yes | no | yes | yes | yes | yes | yes | yes | yes | yes |
| 12 | yes | no | no | yes | yes | no | yes | yes | No | yes | yes | yes |
| 13 | yes | yes | yes | yes | no | yes | no | no | yes | yes | yes | yes |
| 14 | no | no | no | no | no | no | no | no | no | no | yes | yes |
| ITEM | Zhao 2019 | Vohra 2020 | Dalrymple 2018 | Zhao 2017 | Mathias 2010 | Pintada-Palomino 2018 | Zenetti 2019 | Patil 2013 | Theobaldo 2020 | Taraboanta 2019 | Wang 2020 | Kobyashi 2021 |
| 1 | yes | yes | yes | yes | yes | yes | yes | yes | yes | yes | yes | yes |
| 2a | yes | yes | yes | yes | yes | yes | yes | no | yes | yes | yes | yes |
| 2b | no | yes | yes | yes | yes | yes | yes | no | yes | yes | yes | yes |
| 3 | yes | yes | yes | yes | yes | yes | yes | yes | yes | yes | yes | yes |
| 4 | yes | yes | yes | yes | yes | yes | yes | no | yes | yes | yes | yes |
| 5 | no | no | no | yes | no | no | yes | no | no | no | yes | no |
| 6 | no | no | no | no | yes | no | yes | no | no | no | yes | yes |
| 7 | no | no | no | no | no | no | no | no | no | no | yes | no |
| 8 | no | no | no | no | no | no | no | no | no | no | no | no |
| 9 | no | no | no | no | no | no | no | no | no | no | no | no |
| 10 | yes | yes | yes | yes | yes | yes | yes | yes | yes | yes | yes | yes |
| 11 | yes | yes | yes | yes | yes | yes | yes | yes | yes | yes | yes | yes |
| 12 | no | yes | yes | yes | yes | yes | no | yes | no | no | yes | yes |
| 13 | yes | yes | yes | yes | yes | yes | yes | yes | yes | no | yes | yes |
| 14 | no | no | no | no | no | no | no | no | No | no | no | no |

b: Observational studies

| **Reporting quality Assessment modified STROBE statement- observational studies** | | | | | | | |  |  |  |  |
| --- | --- | --- | --- | --- | --- | --- | --- | --- | --- | --- | --- |
|  |  |  |  |  |  |  |  |  |  |  |  |
| ITEM | 1 | 2 | 3 | 4 | 5 | 6 | 7 | 8 | 9 | 10 | 11 |
| Alkhatib 2005 | yes | yes | yes | yes | yes | yes | no | yes | no | no | yes |
| Ness 1977 | no | yes | yes | yes | yes | yes | yes | no | no | no | no |
|  |  |  |  |  |  |  |  |  |  |  |  |
| ITEM | 12 | 13 | 14 | 15 | 16 | 17 | 18 | 19 | 20 | 21 | 22 |
| Alkhatib 2005 | no | no | no | no | yes | no | yes | no | no | yes | no |
| Ness 1977 | no | no | no | yes | no | yes | yes | no | yes | no | no |

c. Randomised control trials

| **Reporting quality Assessment CONSORT - RCT** | | | | |  |  |  |  |  |  |  |  |  |  |  |  |  |  |  |  |
| --- | --- | --- | --- | --- | --- | --- | --- | --- | --- | --- | --- | --- | --- | --- | --- | --- | --- | --- | --- | --- |
|  |  |  |  |  |  |  |  |  |  |  |  |  |  |  |  |  |  |  |  |  |
| ITEM | 1a | 1b | 2a | 2b | 3a | 3b | 4a | 4b | 5 | 6a | 6b | 7a | 7b | 8a | 8b | 9 | 10 |  |  |  |
| Whelton 2012 | yes | yes | yes | yes | yes | n/a | yes | yes | yes | yes | n/a | yes | n/a | yes | yes | no | yes |  |  |  |
|  |  |  |  |  |  |  |  |  |  |  |  |  |  |  |  |  |  |  |  |  |
| ITEM | 11a | 11b | 12a | 12b | 13a | 13b | 14a | 14b | 15 | 16 | 17a | 17b | 18 | 19 | 20 | 21 | 22 | 23 | 24 | 25 |
| Whelton 2012 | yes | no | yes | no | yes | yes | no | no | yes | yes | yes | N/a | no | yes | no | yes | yes | yes | no | yes |
