# Supplementary figures and images for: Effect of tobacco and nicotine in causing staining of dental hard tissues and dental materials: A systematic review and meta‐analysis
Source: Clin Exp Dent Res. 2022 Nov 13;9(1):150–64. doi: 10.1002/cre2.683 (PMC9932248; doi:10.1002/cre2.683)

Supplemental table 7: 11 compounds found in particulate matter (adapted from Haiduc et al 2020)


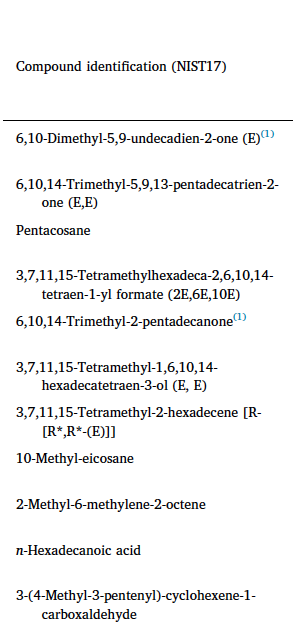

Supplement: Supplementary file 10 — Supplementary information. [file CRE2-9-150-s009.docx]
